# Prostate-specific antigen at or before age 50 as a predictor of advanced prostate cancer diagnosed up to 25 years later: A case-control study

**DOI:** 10.1186/1741-7015-6-6

**Published:** 2008-02-15

**Authors:** David Ulmert, Angel M Cronin, Thomas Björk, Matthew F O'Brien, Peter T Scardino, James A Eastham, Charlotte Becker, Göran Berglund, Andrew J Vickers, Hans Lilja

**Affiliations:** 1 Departments of Laboratory Medicine and Clinical Sciences in Malmö, Lund University, University Hospital UMAS, Malmö, Sweden; 2 Departments of Surgery (Urology), Clinical Laboratories, Medicine (GU-Oncology), Epidemiology and Biostatistics, Memorial Sloan-Kettering Cancer Center, 1275 York Avenue, New York, NY 10021, USA

## Abstract

**Background:**

Based on a large, representative unscreened cohort from Malmö, Sweden, we have recently reported that a single prostate-specific antigen (PSA) measurement at or before age 50 is a strong predictor of prostate cancer occurring up to 25 years subsequently. We aimed to determine whether this association holds for advanced cancers, defined as clinical stage T3 or higher, or skeletal metastasis at the time of the cancer diagnosis.

**Methods:**

In 1974–1986 blood samples were obtained from a cohort of 21,277 men aged up to 50. Through 1999, 498 men were diagnosed with prostate cancer, and of these 161 had locally advanced or metastatic prostate cancers. Three controls, matched for age and date of venipuncture, were selected for each case. Conditional logistic regression was used to test associations between molecular markers and advanced cancer.

**Results:**

Median time from venipuncture to diagnosis was 17 years. Levels of all PSA forms and hK2 were associated with case status. Total PSA was a strong and statistically significant predictor of subsequent advanced cancer (area under the curve 0.791; *p *< 0.0005). Two-thirds of the advanced cancer cases occurred in men with the top 20% of PSA levels (0.9 ng/ml or higher).

**Conclusion:**

A single PSA test taken at or before age 50 is a very strong predictor of advanced prostate cancer diagnosed up to 25 years later. This suggests the possibility of using an early PSA test to risk-stratify patients so that men at highest risk are the focus of the most intensive screening efforts.

## Background

We have previously shown that a single prostate-specific antigen (PSA) measurement taken at age 50 or younger is a strong predictor of prostate cancer diagnosed up to 25 years subsequently [1]. Our study cohort was obtained from the Malmö Preventive Medicine Study, a large, representative, population-based study on cardiovascular risk factors that took place in Malmö, Sweden from 1974 to 1986. Using a case-control design, we found that PSA levels in the archived blood plasma taken during 1974–1986 were significantly higher in men subsequently diagnosed with prostate cancer compared with matched controls. As the rate of PSA screening in Sweden during the study period was very low [2], the study constitutes a 'natural experiment' to test hypotheses about the development of prostate cancer.

Our finding has several implications, most notably that a single PSA test at age 45–50 could risk-stratify the population for the intensity of subsequent screening. However, as is well known, more men die with prostate cancer than from prostate cancer, and many prostate cancers affect neither the length nor the quality of a man's life. For example, autopsy studies of men who die from causes other than prostate cancer have found cancer in the prostates of approximately 20% of men at age 60 and approximately 40% at age 70 [3,4]; this sharply contrasts with the low lifetime risk of dying from this disease. Moreover, in contrast to the very marked country-to-country differences in prostate cancer incidence, there is relatively minor variation in prostate cancer mortality rates [5]. These observations raise the possibility that the sort of cancers predicted by an early elevation of PSA might have little clinical relevance.

Accordingly, we decided to reanalyze our data to focus on an endpoint of unquestionable clinical significance: locally advanced or metastatic disease at the time of prostate cancer diagnosis. Skeletal metastases cause symptoms, such as pain, and most men with metastases die of their disease [6,7]; as regards clinical stage T3, cancer that has demonstrably grown outside the confines of the prostate will, if left untreated, inevitably lead to severe disease symptoms (e.g. local obstruction) or prostate cancer metastasis [8]. In this study, we aimed to determine whether levels of prostate-specific kallikreins (total PSA, free PSA and hK2) measured in anti-coagulated plasma samples could predict locally advanced or metastatic prostate cancer up to 25 years before diagnosis. We also investigated whether combining different markers into a single multivariable model could improve our ability to predict compared to use of total PSA alone.

## Methods

### Study cohort and controls

Subjects, matching and analytical methods of the Malmö Preventive Medicine study have been thoroughly described in previous reports [1]. The Malmö Preventive Medicine study was approved by the Ethics Committee at Lund University and was, in brief, conducted from 1974 to 1986 by inviting men born between 1926 and 1949 to participate in a study intended to primarily investigate risk factors for major cardiovascular and metabolic diseases [9]. EDTA-anticoagulated blood plasma samples were archived at -20° and never thawed until the recent analysis. Our analysis focused on those participants aged 33–50 at baseline, totaling 21,277 (74% of men invited). According to the Swedish Cancer Registry, 498 (2.3%) of these men were diagnosed with prostate cancer up to 31 December 1999. Blood samples were available and analyzable for 462 of these men (93%). In a majority of the cases sextant biopsies were performed and evaluated according to the Gleason system, but some were graded according to World Health Organization (WHO) criteria. Therefore, to obtain a homogenous description of our cohort, we translated all tumor grades into the WHO system. Data on bone scans at the time of diagnosis were available for 370 (80%) of 462 men; clinical stage data were available for 398 (86%).

We used a case-control study design nested within the Malmö Preventive Project cohort. Participants without a prostate cancer diagnosis at the study close date were matched with cases for age and date of baseline venipuncture (± 3 months for both factors for ~95% of controls). Controls were then randomly selected from matches. The present investigation included only those cases with advanced prostate cancer and their respective controls. Advanced prostate cancer was defined as metastases verified by bone scan or clinical stage at least T3 at the time of prostate cancer diagnosis.

There were 161 cases in the study. Although three controls were initially matched for each case, we later found that 47 controls were not followed until diagnosis of the matched case, usually because of early death, leaving 436 controls. Most cases had either two (24%) or three (73%) matched controls; four cases (3%) had one control. Almost all cases (152, 94%) were ages 40–50 at venipuncture. The remaining nine cases were aged less than 40: the ages of each of these patients at venipuncture and at diagnosis were 33/49, 35/53, 35/54, 36/51, 37/57, 37/57, 39/53, 39/59 and 39/59. As age was a matching criterion, the age distribution in controls was very similar.

### PSA measurements

PSA was assayed in anticoagulated plasma stored at -20°C for 17–28 years. Although PSA forms, particularly free PSA, in serum are labile in some storage conditions, we previously showed that the levels of both total PSA and free PSA in anticoagulated plasma are unaffected by long-term storage at -20°C [10]. The levels of total PSA, free PSA and hK2 were measured in archived plasma from cases and controls as described previously [1]. Levels of complexed PSA were calculated as total PSA minus free PSA. Total PSA measured by the assay used, Prostatus Free/Total PSA assay (Perkin-Elmer Life Sciences Turku, Finland), differs by 13% from WHO calibration standards [11].

### Statistical analysis

For our main analysis we defined an event as metastases or clinical stage at least T3 at the time of prostate cancer diagnosis. We conducted conditional logistic regression based on matching for age and date of venipuncture to determine associations between molecular markers and the event. To calculate predicted probability of the event among Swedish men for a given PSA level, we entered PSA in a logistic model using restricted cubic splines with knots at the tertiles. Owing to the 3:1 matched case-control design, the incidence of advanced cancer in our study was close to 25%. To correct the incidence to the expected probability at age 75, we adjusted the probabilities by adding a constant (a Bayes factor) to the linear prediction. The expected probability of advanced cancer at age 75 was derived by taking the estimated probability of prostate cancer by age 75 in Swedish men (10%) [12], and multiplying by the proportion of advancer cancer cases that were observed in this cohort (i.e. number of advanced prostate cancer cases/number of total prostate cancer cases). These probabilities were determined among men aged 44–50 at baseline, as this is just before the age at which men are typically recommended to initiate prostate cancer screening. All statistical tests were two-sided. All analyses were conducted using Stata 9.2 (Stata Corp., College Station, TX).

## Results

There were 161 patients matching the criteria for advanced prostate cancer for our main analysis, 62 with skeletal metastases and 149 with clinical stage at least T3 at the time of prostate cancer diagnosis. Fifty patients had both metastases and clinical stage at least T3. The median time from baseline venipuncture to diagnosis was 17 years (interquartile range 15, 19), with median age at diagnosis 64 years (interquartile range 60, 67). Eighteen patients (11%) had WHO grade I disease, 73 patients (45%) grade II and 69 patients (43%) grade III, with WHO grade missing for one patient. Five (3%) patients were clinically judged to have stage T1 disease, 7 patients (4%) T2 and 149 patients (93%) T3–T4. Twelve (7%) patients had lymph node metastases, 57 (35%) patients did not and 92 patients were not evaluated for nodal status.

**Figure 1:**
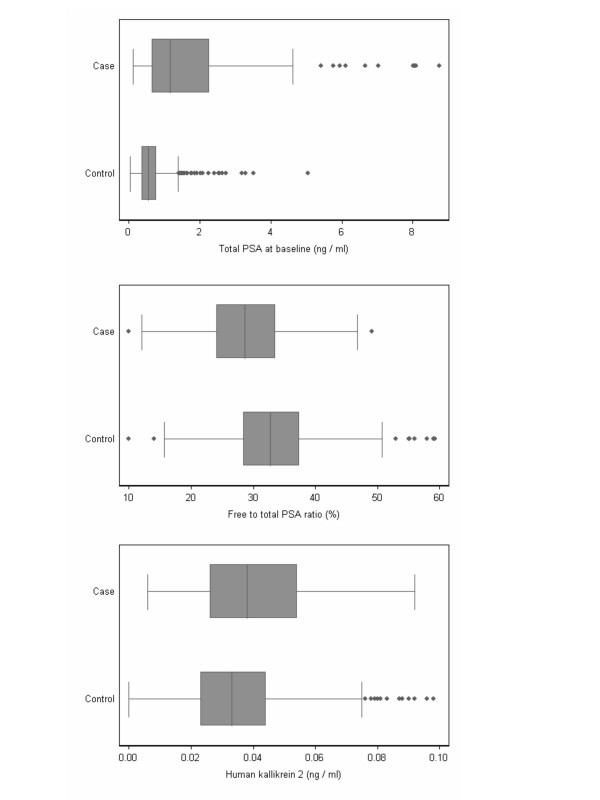
Box plot of total PSA, free-to-total PSA ratio and hK2 levels separately for cases of advanced cancer and controls.

Prostate-specific kallikrein measurements are reported in Table 1 and illustrated in Figure 1. In the anti-coagulated plasma collected at baseline, median levels of total PSA, complexed PSA, free PSA and hK2 were higher, and median free-to-total PSA ratio lower, for men who were found to have skeletal metastases or clinical stage at least T3 at the time of prostate cancer diagnosis, compared with men not diagnosed with prostate cancer.

**Table 1:** Plasma levels of total PSA, PSA subforms and hK2 in cases and controls at baseline venipuncture. Data are given as median (interquartile range). For illustrative purposes, we show data for cases known to be less than T3 and M0 at diagnosis (88 cases had data missing for *either *stage or metastases and were not T3, T4 or M+). As would be expected, controls matched with non-advanced cancers had almost identical markers levels to the controls matched with advanced cases (data not shown).

**Marker**	**Advanced cancer *n *= 161**	**Controls (matched with advanced cases) *n *= 436**	**Non-advanced cancer *n *= 213**
Total PSA (ng/ml)	1.22 (0.67, 2.42)	0.54 (0.36, 0.78)	0.98 (0.61, 1.56)
Free PSA (ng/ml)	0.35 (0.22, 0.67)	0.18 (0.12, 0.26)	0.30 (0.19, 0.45)
Complexed PSA (ng/ml)	0.81 (0.41, 1.64)	0.32 (0.20, 0.51)	0.62 (0.38, 1.04)
Free to total PSA ratio (%)	29.0 (24.2, 33.7)	32.7 (28.3, 37.2)	30.5 (26.2, 33.7)
Human kallikrein 2 (ng/ml)	0.039 (0.027, 0.055)	0.033 (0.023, 0.044)	0.037 (0.028, 0.053)

Table 2 shows the utility of various PSA forms to predict risk of skeletal metastases or clinically advanced disease at the time of prostate cancer diagnosis by univariate analysis. All of the prostate-specific kallikreins were associated with case status (all *p *< 0.0005). An increase of 1 ng/ml in total PSA was associated with an odds ratio for advanced cancer of 4.29 (95% confidence interval (CI) 2.98, 6.18). Odds ratios from the multivariable model are not presented here because of high collinearity between the markers: for example, the correlation between free and total PSA was 0.963. To assess whether markers other than total PSA could aid in discrimination of advanced cancer (skeletal metastases or clinical stage at least T3 at diagnosis) from no cancer diagnosis, we calculated the area under the curve (AUC) for all markers alone using ten-fold cross-validation. Total PSA (AUC 0.791) and complexed PSA (AUC 0.793) discriminated advanced cancer from no cancer most strongly. We then fitted a multivariable model consisting of total PSA, free PSA, free-to-total PSA ratio and hK2; the AUC for this model was 0.785. Hence, there was no evidence that the additional markers added discriminative accuracy above that of total PSA alone. We therefore focused on total PSA for the remaining analyses.

**Table 2:** Univariate associations between molecular markers and advanced prostate cancer at the time of diagnosis. Advanced cancer was defined as skeletal metastases or clinical stage ≥ T3. Predictive accuracy of each marker is given as AUC. To facilitate comparisons between markers, the odds ratios are for an increase of one-quarter of a standard deviation.

**Marker**	**Odds ratio**	**95% CI**	**AUC**
Total PSA	3.19	2.38, 4.27	0.791
Free PSA	3.06	2.30, 4.08	0.776
Complexed PSA	3.11	2.33, 4.16	0.793
Percent free to total PSA	0.85	0.80, 0.90	0.641
Human kallikrein 2	1.25	1.13, 1.38	0.604

Small increases in total PSA markedly increased the risk of a subsequent diagnosis of advanced prostate cancer (Table 3). For example, a modestly increased total PSA level of 1.01–2 ng/ml raised the odds more than seven-fold compared with a total PSA of 0.5 ng/ml or less, and the odds increased 22-fold for a total PSA 2.01–3 ng/ml. To ensure that our results are not explained purely by events occurring shortly after venipuncture, we performed additional analyses in subgroups based on time from venipuncture to diagnosis (Table 4). Total PSA was a predictor of advanced disease among men diagnosed 16–19 and 20 years or more following venipuncture (both *p *< 0.0005). There was an apparent increase in odds ratio for diagnosis after 20 years. We believe that this is a chance finding related to chance differences in controls; the mean total PSA in controls for patients diagnosed more than 20 years following venipuncture was 0.56 ng/ml compared with 0.69 ng/ml in controls for patients diagnosed less than 20 years after venipuncture. Hence, although our data support the hypothesis that total PSA can predict advanced prostate cancer at the time of diagnosis up to 25 years later, we do not believe they can be used to suggest that total PSA predicts later cancers more effectively than earlier cancers.

**Table 3:** Odds of advanced cancer at the time of diagnosis related to different levels of total PSA

**Total PSA (ng/ml)**	**Cases**	**Controls**	**Odds ratio**	**95% CI**
0.00–0.50	22	204	Reference	-
0.51–1.00	42	165	2.37	1.33, 4.23
1.01–2.00	45	55	7.25	3.89, 13.5
2.01–3.00	25	10	21.5	8.58, 53.8
3.01+	27	2	120	26.0, 557

**Table 4:** Odds ratio for advanced prostate cancer diagnosis, calculated according to delay from baseline venipuncture to diagnosis. Odds ratios are for each 1 ng/ml increase in PSA.

**Delay to diagnosis**	**Cases**	**Odds ratio**	**95% CI**	***p*-value**
≤15 years	59	4.30	2.40, 7.72	<0.0005
16–19 years	63	3.05	1.85, 5.04	<0.0005
≥20 years	39	11.0	3.09, 38.9	<0.0005
All	161	4.29	2.98, 6.18	<0.0005

Similar results were found for the other kallikreins: all but hK2 (*p *= 0.06) remained statistically significant predictors of advanced prostate cancer among men diagnosed at least 20 years after venipuncture.

**Figure 2:**
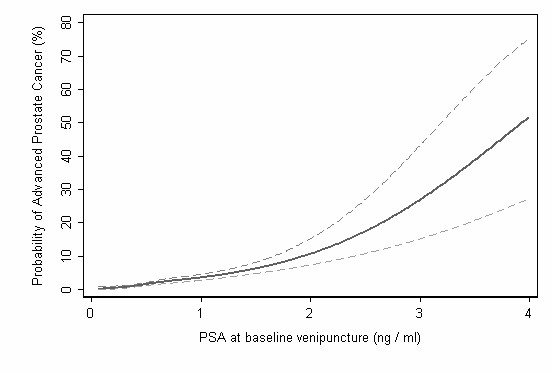
Predicted probability of advanced prostate cancer by the total PSA level in anti-coagulated plasma measured at age 44–50. Dashed lines indicate the 95% CIs.

**Figure 3:**
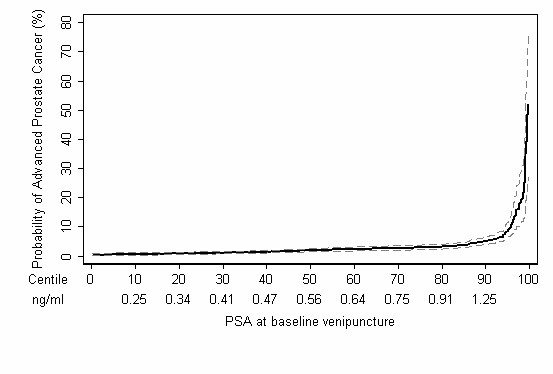
Predicted probability of advanced prostate cancer by population-based centiles of PSA measured at age 44–50. Dashed lines indicate the 95% CIs.

Using the Bayesian approach described above, we estimated the probability of advanced prostate cancer at the time of diagnosis for different levels of total PSA in the plasma obtained from the 142 cases (88%) and 382 controls who were ages 44–50 at baseline venipuncture (Figures 2 and 3). For those with total PSA 0.5–1 ng/ml, the risk of advanced prostate cancer was 2–4%, close to the population mean risk of 3.5%. Subjects with a total PSA of 2 ng/ml had a risk of advanced prostate cancer of 12%, more than three times the population mean. Sixty-nine (49%) of advanced cancers occurred among the 10% of subjects with the highest PSA levels (total PSA at least 1.2 ng/ml), while 94 (66%) occurred among the top 20% (total PSA at least 0.9 ng/ml).

Our results were based on the pre-specified definition of advanced prostate cancer (skeletal metastasis or clinical stage at least T3 at the time of prostate cancer diagnosis). To check whether our results were sensitive to the definition of advanced cancer used, we performed additional analyses with two different definitions. The first was more inclusive, defining advanced prostate cancer as any of skeletal metastasis, clinical stage at least T3, lymph node involvement or WHO grade III at the time of prostate cancer diagnosis. The second was less inclusive and considered only presence of skeletal metastasis at diagnosis. This did not have a major effect on any of the results: some key statistics are shown in Table 5 for each definition of advanced prostate cancer.

**Table 5:** Sensitivity analyses

		**Univariate odds ratio (95% CI)**		**Predictive accuracy (AUC)**		
		
**Definition of advanced cancer**	**Number of cases/controls**	**Total PSA (ng/ml)**	**Free to total PSA (%)**	**Total PSA (ng/ml)**	**Free to total PSA (%)**	**Multivariable model**
Metastases or ≥ T3	161/436	4.29 (2.98, 6.18)	0.92 (0.89, 0.95)	0.791	0.641	0.785
Less inclusive*	62/163	3.02 (1.87, 4.90)	0.90 (0.84, 0.95)	0.762	0.674	0.751
More inclusive†	190/512	4.05 (2.89, 5.67)	0.92 (0.89, 0.95)	0.782	0.645	0.780

* Presence of skeletal metastases at time of diagnosis.

† Presence of any of the following criteria at time of diagnosis: skeletal metastasis, clinical stage at least T3, positive lymph node, WHO grade III.

## Discussion

The Malmö Preventive Medicine cohort was enrolled before the PSA era, and there was no subsequent recommendation for prostate cancer screening in this region. The rate of PSA testing has accordingly remained very low up to our current study endpoint [2]. Furthermore, the participation rate (74%) was high and case ascertainment was complete owing to the excellent coverage of the Swedish National Cancer Registry [13]. Our study is therefore not subject to the sort of verification and selection biases commonly associated with prostate cancer studies and provides a 'natural experiment' to test hypotheses concerning the long-term prediction of prostate cancer.

Our results describe the relationship between prostate kallikreins in blood plasma obtained at a single occasion at age 50 or below and the diagnosis of advanced prostate cancer up to 25 years later. We found that prostate-specific kallikreins were significantly increased decades before the clinical manifestation of advanced disease. The predictive accuracy of total PSA was very high (AUC 0.791). Modestly increased levels of total PSA in the ranges of 1.01–2 ng/ml and 2.01–3 ng/ml were associated with 7- and 22-fold elevated odds of advanced prostate cancer, respectively. The majority of advanced cancers (66%) occurred in the 20% of the population with the highest PSA levels. It is also noteworthy that our study of archived blood samples is taken from a highly representative, population-based sample. There was no selection based on test results (indeed, this would not have been possible, as the PSA test was not available during the period when bloods were drawn). As such, we can be confident that our reported test characteristics reflect those of the population to which we would like to apply our results.

These results confirm our previous finding of an association between PSA levels and subsequent prostate cancer, and suggest that this association is not restricted to cancers unlikely to affect a man's survival or quality of life. Indeed, we found total PSA to be more strongly predictive of subsequent advanced prostate cancer (AUC 0.791) than of any prostate cancer (AUC 0.762). This illustrates the rule of thumb that it is easier to predict more extreme medical events. Free-to-total PSA ratio and hK2, two markers associated more specifically with malignancy, were far less predictive than total PSA, a marker associated with both benign and malignant prostate conditions. This suggests that PSA elevations in cases below or at age 50 may be related to a premalignant state, or to a carcinogenic process, rather than the presence of malignant cells in the prostate.

A possible limitation of our study is that any definition of clinically significant prostate cancer is open to question. However, we repeated our analysis altering our definition of advanced cancer to be more restrictive (only patients with skeletal metastases at diagnosis) or more inclusive (skeletal metastases or clinical stage al least T3 or positive lymph nodes or grade III at time of diagnosis) and found no important differences in our results. It might also be argued that death from prostate cancer would be the most appropriate endpoint, but owing to a small number of events in our current study cohort, statistical analysis would have been underpowered. Future studies will address the relationship between kallikreins and death from prostate cancer as the cohort matures.

Previously, both our group and other investigators have studied the association between PSA level in the blood and the long-term risk of being diagnosed with any stage of prostate cancer, but did not specifically address whether these findings were applicable to diagnosis of advanced tumors [1,14-16]. Our findings reconfirm previous analyses suggesting that prostate cancer can be predicted many years before it is diagnosed and that we might therefore reduce prostate cancer mortality by intervening at an early stage when curative treatment is still possible.

Current US prostate screening guidelines recommend that all men over age 50 who have a life expectancy of at least 10 years should have an annual digital examination and PSA test. Results from ongoing screening trials show that these recommendations result in over-diagnosis and over-treatment. For example, the Rotterdam section of the European Randomized Screening Study for Prostate Cancer have shown that almost 50% of screen-detected cancers are indolent (organ-confined, Gleason 6 or less, and 0.5 cc or less in volume) and thus unlikely to affect a man's survival or quality of life [17]. Over-treatment is associated not only with high morbidity, such as poor erectile, bowel and urinary function resulting from surgery or radiotherapy, but also high healthcare costs. A screening program that focused on those at highest risk of prostate cancer morbidity or mortality might well have a superior benefit-to-harm ratio compared with the current approach of screening all men. One scenario might be to make exceptional efforts to ensure that all men obtain a PSA test in their mid to late forties. Although a man with a PSA elevated above the threshold for biopsy (e.g. 3 ng/ml) could be referred for immediate biopsy, this would be very rare (~1–2% in the current data set); the primary purpose of the early PSA test would be to determine which men should be invited back for regular screening at age 50 and which men advised that PSA screening is unlikely to benefit them.

This study is based on a previously published case-control study in which we attempted to predict the occurrence of prostate cancer at any stage. We did not re-match for this study and hence patients with a prostate cancer diagnosis were not included in the sample from which controls were selected. We note that kallikrein levels of these 301 participants are likely to be higher than those of Malmö Preventive Medicine participants not diagnosed with prostate cancer, thus inflating differences between cases and controls in our current analysis. However, the original Malmö Preventive Medicine cohort contains approximately 21,270 participants who could act as controls, from whom 436 controls were randomly chosen to be included in our current analysis. As there were 462 prostate cancer cases, we would expect prostate cancer patients to comprise approximately 2% of the control group. Therefore, we conclude that our failure to sample from non-advanced prostate cancer cases is unlikely to have any major effect on the current results.

## Conclusion

A PSA level measured at a single occasion in blood drawn at age up to 50 is a very strong predictor of being diagnosed with advanced prostate cancer up to 25 years later. This raises the possibility that screening for prostate cancer could be risk-stratified so that men at highest risk are the focus of the most intensive screening efforts.

## Abbreviations

AUC, area under the receiver operating characteristic curve; EDTA, ethylenediamine tetraacetic acid; PSA, prostate-specific antigen; WHO, World Health Organization

## Competing interests

Dr Hans Lilja holds patents for free PSA and hK2 assays.

## Authors' contributions

HL is the principal investigator and was responsible for the study design had full access to all data and data analyses, and had final responsibility for the decision to submit the manuscript. GB was mainly responsible for the study cohort, the case-control design nested within the study cohort. DU, CB and HL were responsible for measuring PSA forms and hK2. DU and TB were responsible for reviewing the patient medical records. AMC and AJV were responsible for all biostatistical analyses and workup. MFO, JAE and PTS actively contributed to all elements of the study. All authors read and approved the final manuscript.

## Pre-publication history

The pre-publication history for this paper can be accessed here:


